# The Physics and Manipulation of Dean Vortices in Single- and Two-Phase Flow in Curved Microchannels: A Review

**DOI:** 10.3390/mi14122202

**Published:** 2023-12-01

**Authors:** Yeganeh Saffar, Sina Kashanj, David S. Nobes, Reza Sabbagh

**Affiliations:** Mechanical Engineering Department, University of Alberta, Edmonton, AB T6G 2R3, Canada; ysaffar@ualberta.ca (Y.S.); kashanj@ualberta.ca (S.K.); david.nobes@ualberta.ca (D.S.N.)

**Keywords:** Dean flow, curved microchannel, particle sorting, droplet deformation

## Abstract

Microchannels with curved geometries have been employed for many applications in microfluidic devices in the past decades. The Dean vortices generated in such geometries have been manipulated using different methods to enhance the performance of devices in applications such as mixing, droplet sorting, and particle/cell separation. Understanding the effect of the manipulation method on the Dean vortices in different geometries can provide crucial information to be employed in designing high-efficiency microfluidic devices. In this review, the physics of Dean vortices and the affecting parameters are summarized. Various Dean number calculation methods are collected and represented to minimize the misinterpretation of published information due to the lack of a unified defining formula for the Dean dimensionless number. Consequently, all Dean number values reported in the references are recalculated to the most common method to facilitate comprehension of the phenomena. Based on the converted information gathered from previous numerical and experimental studies, it is concluded that the length of the channel and the channel pathline, e.g., spiral, serpentine, or helix, also affect the flow state. This review also provides a detailed summery on the effect of other geometric parameters, such as cross-section shape, aspect ratio, and radius of curvature, on the Dean vortices’ number and arrangement. Finally, considering the importance of droplet microfluidics, the effect of curved geometry on the shape, trajectory, and internal flow organization of the droplets passing through a curved channel has been reviewed.

## 1. Introduction

Microfluidic devices have been continuously developed since the invention of the first laboratory on-chip (LOC) in 1979 [1,2]. They have been developed to manipulate and control the flow and its contained materials and features, such as cells, capsules, and particles [3,4,5,6]. Employment of the manipulation devices is mostly preferred in comparison to others such as flow cytometry, centrifuge, and membrane-based filtering schemes, due to the simple structure of the channels and their high throughput [7,8,9,10]. This method requires less of the solution sample and is cost effective, with a higher efficiency [7,8,9,10]. Many microfluidic devices have been invented for a wide range of applications, since bio-cells, particles, and capsules behave differently based on their physical properties during manipulation [11]. Detecting malaria-infected blood cells [12], circulating tumor cells (CTCs) [13], and measuring glucose concentration using various bio fluids [14,15] are examples of diagnostic applications. Controlled chemical reactions [16], protein expression [17], and organic and non-organic compound synthesis [18,19] are among the chemical applications of microfluidic devices. There are also several other applications in the cosmetics [20,21,22], food [23,24,25,26], agriculture [27,28,29], and pharmaceutical [30,31,32] industries, manipulating flows and their contents at the micro-scale.

Based on the implementation of an external source of energy, manipulation methods in microfluidics can be passive or active [33,34,35]. Geometrical manipulation is a passive method that utilizes the microchannel’s geometric properties to control the flow [36,37]. For example, channels with grooves form stream-wise vortices which can be employed for mixing enhancement [38,39,40,41]. T-junction channels generate monodisperse droplets which are a method of cell and particle encapsulation [42,43]. Curved microchannels are other examples of passive manipulation devices that can be used for particle separation [44], cell sorting [45], and mixing purposes [46]. The asymmetric shape of a curved microchannel wall induces a type of secondary flow, counter-rotating vortices, called Dean vortices, and such a flow is called the Dean flow or secondary flow [47]. In mixing devices, these vortices increase the mixing efficiency and manipulate the behavior of particles and droplets by changing their equivalent positions and affecting their topology [48,49]. 

Considering the ongoing research interest on the manipulation of micro-scale flow behavior, several review papers have been published in this area, discussing the Dean flow [50]. Zhang et al. [11] presented a review paper that discusses the fundamental kinematics of particles and a comprehensive review of recent developments in inertial microfluidics. They briefly introduce Dean vortices in a spiral channel and the particle sorting application benefiting from this geometry [11]. Zhao et al. [51] reviewed the application of the secondary flow generated by different single-layer and multilayer geometrical designs, e.g., microchannels with obstructions, spirals, serpentines, and double-layered with a groove array. In their paper, the applications of the Dean flow as a type of secondary flow are reviewed as well. Afsaneh and Mohammadi [52] presented a review on the manipulation of cells and particles [52]. They have discussed the Dean flow briefly, in a curved channel, as a fluid-based manipulation method [52]. Recently, Mishra et al. [53] briefly reviewed the physics of the Dean flow with a primary focus on biomedical applications. There are also some papers extensively reviewing the effect of the Dean flow and curved microdevices on the sorting and manipulation of particles [11,54]. Hence, these topic areas are not covered in this review.

There are many works investigating the Dean flow in microchannels to provide a better understanding of the physics of the phenomena, the effect of the geometrical properties on the vortices, and the interactions between Dean vortices and capsules. Although the Dean flow has been mentioned briefly in some review papers, there is no literature review concentrating on this phenomenon specifically at the micro-scale. The lack of sufficient information about the Dean flow at the micro-scale precludes researchers from performing physics-based design and optimization. Understanding the physics of the Dean flow in different operating conditions at the micro-scale provides the knowledge required to optimize the working window of a microfluidic device based on physics.

**Figure 1:**
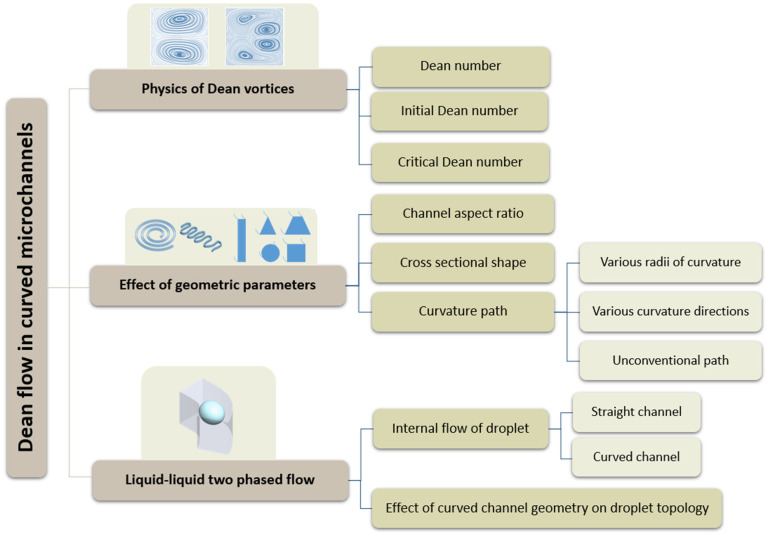
Schematic diagram of the paper indicating the layout of the discussion.

This review is focused on three substantial areas that are influenced by Dean vortices, which form the organization of this work, as shown in Figure 1. The phenomena of Dean vortices and their flow structure are reviewed in the first section. The second section discusses the relationship between the generated Dean vortices and the geometric properties of a curved microchannel such as the cross-section aspect ratio, cross-section shape, and potential 3D path of the channel. The final section reviews studies investigating the interaction between Dean vortices and capsules in curved microchannels. How the deformation and trajectories of the capsules, and the topology of the capsules’ internal flow, are affected by Dean vortices is reviewed in this section as well.

## 2. The Physics of Flows in a Curved Channel

**Figure 2:**
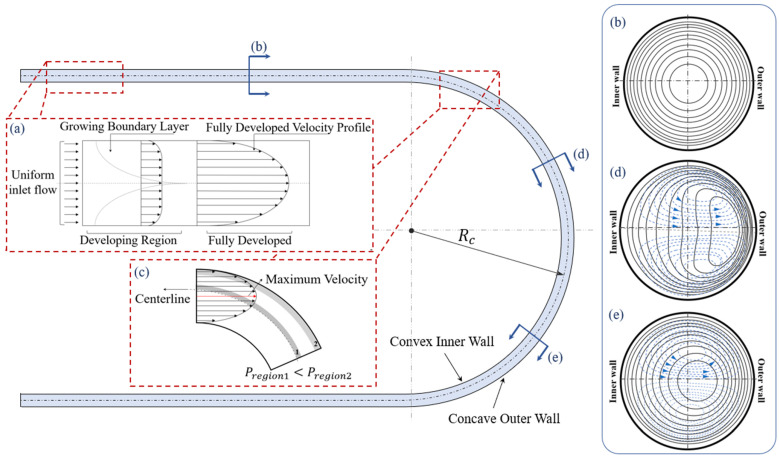
Flow in a curved channel: (**a**) velocity profile of the developing and developed flow in a straight channel far from the curved section, (**b**) constant velocity lines of the flow in the straight section of the channel, (**c**) unsymmetrical velocity profile of the Dean flow entering the curved section of the channel, (**d**) constant velocity lines of the flow in the entrance of the curved section, (**e**) further into the curved section where the center of the vortices are shifted more toward the concave wall.

The velocity profile and flow structure of the Poiseuille flow in a channel with a circular cross-section and an initial radius of curvature $R_{c}=\infty$ (straight channel) is shown in Figure 2a [55,56]. In the straight section, the velocity profile is axisymmetric and the maximum velocity point is on the centerline of the channel [57,58]. Circular constant velocity contours in a channel with a circular cross-section are concentric with the channel cross-section, as shown in Figure 2c [57,58]. The symmetrical shape of the channel walls and cross-section balances the velocity and pressure gradients and generates a symmetrical flow diagram [57,58].

Berger et al. [59] highlighted that investigations into the physics of the flow inside curved channels began in the early 1900s. In 1902, an experimental observation in a curved pipe determined that the location of the maximum velocity moves toward the concave outer wall [60]. Later, Eustice (1910, 1911) injected ink into water passing through a pipe and used its streamline motion to demonstrate the existence of the secondary flow [61,62]. In 1928, for the first time, Dean [47,63] realized that in a pressure-driven system, the flow rate slightly decreases when increasing the channel curvature’s radius. For low velocity flows in a curved channel with the Reynolds number Re=ρUd/μ<2000, where ρ is fluid density, U is the uniform velocity, d is the hydraulic diameter of the channel, and μ is the dynamic viscosity, it was proposed that the radius of curvature is proportional to variations in the flow rate through the parameter, K, defined as:

(1) K=2Re2dRc

In this equation, d is the hydraulic diameter of the channel and $R_{c}$ is the average of the radius of the curvature of the walls [47].

A fully developed flow entering a curved channel develops a centrifugal force in an asymmetrical geometry [64]. Such asymmetricity affects the parabolic velocity profile and causes a shift in the location of the maximum velocity compared to a straight microchannel [65]. Therefore, the maximum velocity shifts from the centerline toward the concave outer wall and forms an asymmetric velocity profile [59,66], as depicted in Figure 2c. This velocity profile induces a high pressure difference between the location of maximum velocity and the concave wall as $P_{region2}>P_{region1}$ [56,67]. This induced pressure gradient results in a transverse flow motion on the channel centerline. As a result of this transverse motion, a secondary flow is formed in the flow field [56,65,67]. The secondary flow causes an energy loss from the main flow stream in the curved channel, which increases the required pressure difference for a certain flow rate compared to a straight channel with the same cross-sectional area. As a result, with a constant pressure difference between the inlet and outlet of a channel, the flow rate in the curved channel will be less than that of the straight channel [47,56,65,67]. 

In sections (d) and (e) of the curved channel of Figure 2, flow streamlines and constant velocity contours in the cross-section of a curved channel are shown [68,69]. With an increase in the velocity, the constant velocity contour lines, shown by the solid lines in Figure 2b, start to deform from circles into bended ovals leaning towards the concave outer wall. The flow streamlines show that the secondary flow pattern forms as two symmetrical and counter-rotating vortices on the top and bottom of the channel [59,69]. These vortices are known as the primary Dean vortices [59,69]. It is shown that, for a higher velocity, the pressure gradient between the slow-pressure zone close to the concave outer wall and the high-pressure zone close to the convex inner wall is higher [47,59,69]. Consequently, as the velocity increases the center of the vortices shifts towards the concave outer wall [69].

In terms of the scale, the principles of the Dean flow remain the same for micro- and macroscales. However, the specific details and considerations may differ. At the micro-scale, such as in microfluidics, the channels typically have dimensions in the order of tens to hundreds of micrometers [70,71]. At such a scale, the flow characteristics in the microchannel may exhibit unique behaviors due to the dominance of viscous forces over inertial forces [49,72]. Consequently, the Reynolds number (the ratio of inertial forces to viscous forces) may be much lower in microchannels [65]. At the macroscale, such as in larger channels or pipes, the Dean flow is still present. However, the relative importance of inertial forces increases compared to the viscous forces. Thus, the Reynolds number is typically higher at the macroscale, and inertial effects play a more significant role in the dynamics of the flow [49]. 

### 2.1. The Dean Number

The non-dimensional parameter, K in Equation (1) was introduced by Dean [47] to investigate the effect of channel curvature on flow. This parameter was later named after its founder as the Dean number, De [59]. This dimensionless number indicates the relationship between the channel geometry and the reduction in flow rate in curved channels [59]. In addition to Dean’s definition in Equation (1), there are several other variations of this equation developed by other researchers. Table 1 shows the various equations for the Dean number that have been used in experimental studies and reported in the literature. 

Theoretical investigations typically deploy either the mean velocity of the channel or pressure gradient as the driving factor to calculate the Dean number. In experimental studies, using the mean velocity is more common since it is more convenient to measure velocity than the pressure gradient, considering the fact that, in complex flow geometries, the pressure gradient changes in different directions. However, for a fully developed flow, the differences between calculations based on the mean axial velocity and pressure gradient are minor [59]. Therefore, in numerical studies, the pressure gradient is usually used instead of the mean velocity estimation to reduce the uncertainty of the calculations [61]. 

The inconsistency in the definition of geometrical parameters such as the measure of the radius of curvature based on the convex wall, concave wall, or centerline of the channel, is another reason for the various definitions of *De*. Thus, it is always important to identify the equation that each study has used to calculate the De since in a similar case using a different definition can cause disagreement between the results. Comparing the results from different studies without considering the calculation references can lead to incorrect conclusions. As a result, it is necessary to study the definitions of *De* and their differences based on their applications.

Most of these equations use similar parameters, such as the Reynolds number, Re, based on the hydraulic diameter of the channel and its inlet velocity; $R_{c},$ the radius of curvature; and d, the width of the channel, in their definitions. But they are slightly different, with an additional constant coefficient or a constant power. The equation introduced by Dean will be referred to as K in this paper and the other equations will be compared to it, with K as the basic equation.

In Table 1, Equation (2) is the extended form of (1), K, represented in this paper [63]. Equation (3) uses the inlet and outlet mass fluxes as defining parameters to calculate the Dean number. The calculated Dean number in this case has an inverse relation with d, which results in a different trend in cases where the channel width changes from the basic equation. Equation (4) is proposed for a constant-radius pipe, so d is assumed to be constant, which results in $De_{2}={\left(K/2\right)}^{\frac{1}{2}}$. Equation (5) is the square root of the K multiplied by $\sqrt{2}$ ($De=\sqrt{2K}\;$). Equation (6) is used cases with a high radius of curvature, such that the convex wall radius of curvature, $r_{i}$, and the concave wall radius of curvature, $r_{o}$, are assumed to be equal (riro≅1) in this scenario; instead of the radius of curvature of the centerline, the convex wall radius of curvature is used. Equation (7) is similar to (5) $De_{1}={\left(2K\right)}^{\frac{1}{2}}/2$ but instead of the centerline radius of curvature, the convex wall radius of curvature is applied. Equation (8) is observed to be the most common equation in experimental investigations, which is related to K as De=K41/4 .

Table 2 contains some of the Dean number definitions obtained in most cases from numerical studies that use the pressure gradient as a part of their definitions. In these equations, G is the pressure gradient, defined as $G=\partial P/\left(R\partial \theta \right)$, where θ is the angular direction in cylindrical coordinates, and $C=Gd^{2}/\left(\mu W_{0}\right)$ is a dimensionless constant. Similar to the equations defined for the experimental studies, these equations can be derived from each other. As an example, modifying (11), considering a constant pipe radius and pressure gradient leads to the simplification shown in (10) [59]. In fully developed flow cases where the pressure gradient can be assumed constant throughout the channel, the Dean number is less sensitive to the application of the mean velocity or pressure gradient. In such cases using both categories would provide a similar result.

### 2.2. Dean Number Thresholds

**Figure 3:**
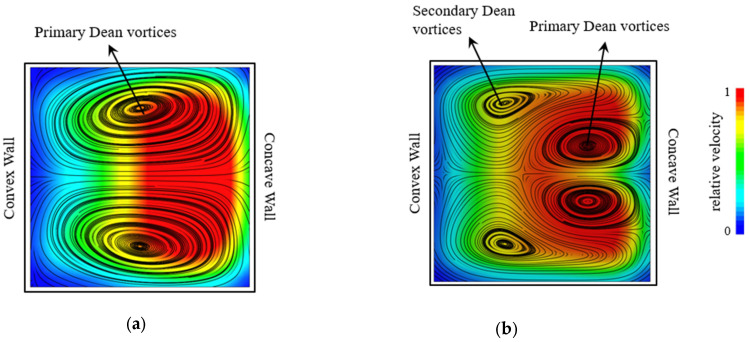
Visualization of the out of plane velocity contours on the background and Dean vortices shown by streamlines inside a rectangular channel. (**a**) Formation of the primary Dean vortices. (**b**) Formation of the secondary Dean vortices.

Various dominant or temporary configurations of Dean vortices can be formed by changing the flow or geometric parameters. The appearance of these configurations can be characterized by the Dean number [66,79]. When increasing the Dean number from 0, the first configuration of Dean vortices, a pair of counter-rotating vortices, appears and are called the primary Dean vortices. An example of these is shown in Figure 3a [64,65], in a radial cross-section view of a curved channel with a rectangular cross-section. The first Dean number threshold in which the vortices appear for the first time is termed the initial Dean number, $De_{i}$. Increasing the Dean number such that $De_{i}<De$ leads to an increase in the transverse velocity of the flow and the strength of the vortices generated. This can be achieved by increasing the flow rate such that the maximum velocity location moves further toward the concave wall, as is depicted in Figure 3b. As a result, the core of the Dean vortices shifts to the concave wall [64,65]. With further increasing of the Dean number, two secondary Dean vortices detach from the main vortices. These secondary vortices are smaller than the main/primary vortices. Figure 3b shows a flow configuration including primary and secondary Dean vortices. The Dean number at which the secondary Dean vortices start to form is called the critical Dean number, $De_{c}$ [65,80,81].

**Figure 4:**
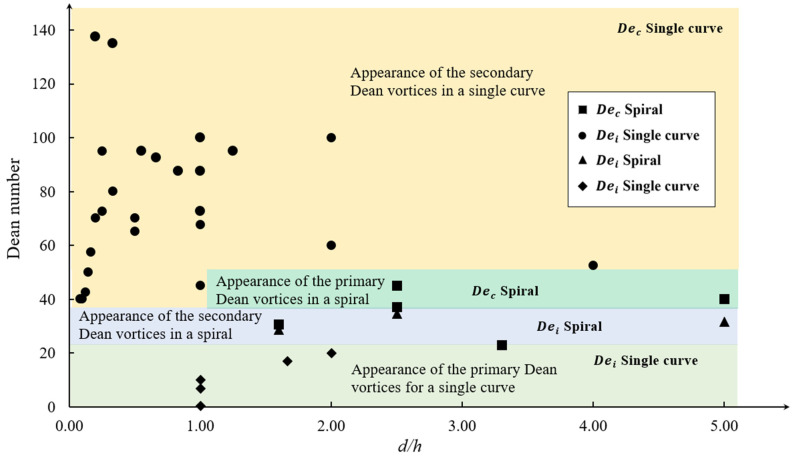
Critical and initial Dean numbers in single and multiple curved geometries, demonstrating the effect of the channel shape in addition to other geometrical properties [55,65,77,80,82,83,84,85,86,87,88].

The values of the initial and critical Dean numbers depend on various factors such as the aspect ratio (d/h), which is defined as the ratio of the channel width or diameter, d, to the height, h, and the radius of curvature, $R_{c}$ [65,79]. Being a function of several parameters makes it difficult to predict the flow configuration in a geometry at a given flow state without numerical or experimental investigations. The values of the Dean numbers, which are all recalculated based on (8), and the channel cross-sectional aspect ratios for different curve and spiral microchannel geometries have been derived from the literature and presented in Table 3. These values are used to generate the phase map shown in Figure 4, which compares De against the channel aspect ratio, d/h. The color variance in the map differentiates the regions of appearance of different Dean vortices. It can be determined from this figure that in a single curve geometry with various channel widths or radii of curvatures, as shown in Figure 3, the initial Dean number is in the range of $0<De_{i}<20$, while the critical Dean number is in the range of $100<De_{c}<160$. For spiral microchannels, the range is significantly different for the initial and critical *De* than the single curve channel case. Both the initial and critical Dean numbers for spiral cases are close to each other, in the range of $20<De_{i}<50$.

It is shown in Figure 4 that the critical and initial Dean number (using the same Dean number definitions for all data sets) for a spiral geometry are located in a very small range in the low Dean number area, which indicates a small window for the appearance of two primary Dean vortices. The initial Dean number of a single curve is in the same area and about half of the initial Dean number of a spiral. This means that the Dean vortices can be generated at lower velocities in a single curve. The critical Dean number for a single curve is found to be in the high Dean number area and almost three times bigger than the initial Dean number for the same geometry.

In reviewing the variables included in the Dean number for the definitions outlined in Table 1 and Table 2, it is observed that the channel shape parameters are not considered defining parameters. This is despite the fact that changing the channel shape has a significant effect on the critical and initial Dean number, as shown in Figure 4. Hence, it can be understood that in addition to the basic geometric parameters, parameters related to the shape of the curved channel are also important and affect the formation of Dean vortices. This is an important outcome and highlights the need for further investigations on the effect of geometry and the inability of the available correlations to predict the flow behavior in curved microchannels and the formation of secondary Dean vortices. The following section looks extensively at the literature on the effect of geometry on the Dean flow.

## 3. The Effect of Geometry on the Structure of the Dean Flow

The formation and topology of Dean vortices are related to both flow properties, such as flow velocity and fluid viscosity, and channel geometry, as can be seen in Equation (1). In addition to the curvature ratio, $d/R_{c}$, that directly affects the Dean number, there are other factors that can affect the velocity field in Dean flows [77,79]. Geometry features such as the channel cross-sectional shape; aspect ratio; and the curvature path, which is the shape of the channel centerline indicating the layout of the channel, are some of those factors. These factors affect the formation of Dean vortices by increasing the initial or critical Dean number, changing the shape of vortices, and changing the direction of rotation [39,46,89,90,91]. Manipulating the specifications of the Dean vortices with geometry can lead to the design of more efficient microchannels for various applications [83]. There are a few studies investigating flow properties directly [38,92]. Studies on applications such as mixing and encapsulating aid our understanding of the effect of the channel’s geometry on the formation and properties of Dean vortices. This section discusses the research results that help us to understand the effect of the channel cross-section shape, aspect ratio of the cross-section, and curvature path, on the Dean vortices.

### 3.1. Effect of the Chanel Aspect Ratio

**Figure 5:**
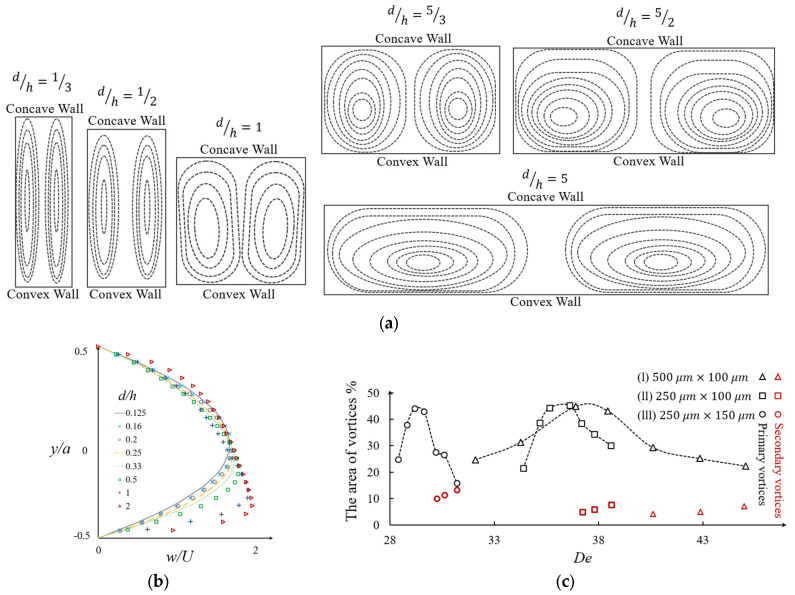
(**a**) Evaluation of the Dean vortices according to the aspect ratio, d/h = [1/3, 1/2,1, 5/3, 5/2, 5] after [77]; (**b**) axial velocity profiles for aspect ratios in the range of 0.125 ≤ d/h ≤ 2 with non-dimensional channel width [79]; (**c**) the evaluation of the Dean vortices’ area as a percentage of channel cross-sectional area with respect to the Dean number, *De*, for different aspect ratios: (I) d/h = 5/3, (II) d/h = 5/2, and (III) d/h = 5 [65]; red dots represent the secondary Dean vortices.

Rectangle and square are the most conventional cross-sectional shapes that have been used in microchannels [74,77,79]. The aspect ratio of the rectangle is a geometric factor that affects the lateral flow velocity, Dean vortices, and their topology [77]. Several studies have focused on the effect of the aspect ratio on Dean vortices and the performance of microdevices [11]. Norouzi and Biglari [77] investigated the effect of the aspect ratio of a rectangular curved duct on the Dean vortices by applying an analytical perturbation solution. The results showing the effect of this ratio are in Figure 5a, with d/h = 0.25 to 4 as the Reynolds number varies from 0 to 100 [77]. Their analytical solution showed that increasing the aspect ratio leads to a decrease in the critical Dean number, $De_{c}$ [77]. It can be inferred that decreasing the aspect ratio leads to a decrease in the lateral velocity of the flow. This implies that by decreasing the aspect ratio and moving from a square cross-section to a high aspect ratio, the mixing efficiency will be increased [80]. 

In a similar work, Fellouah et al. [79] numerically determined the non-dimensional axial velocity profile over a non-dimensional channel width, as shown in Figure 5b. For aspect ratios in the range of 0.125 ≤ d/h ≤ 2, increasing the aspect ratio of the microchannel forces the maximum velocity location to move toward the concave wall of the channel [79]. Also, the maximum velocity value increases by increasing the aspect ratio of the channel cross-section. 

Another way to study the development of vortices in different aspect ratios is using the projected area occupied by the Dean vortices. This method is mostly used in experimental mixing studies where one of the streams is visualized using a dye. Figure 5c represents the evaluation of the projected cross-sectional area of Dean vortices as a percentage of the channel cross-sectional area, with the associated Dean numbers in different aspect ratios of d/h = 5/3, 5/2, and 5 [65]. In Figure 5c-(I), where the aspect ratio is d/h = 5/3, it can be seen that the projected area of the Dean vortices is less than 45% for most Dean numbers except for De = 29. By increasing the aspect ratio to d/h = 5/2, as shown in Figure 5c-(II), the projected area increases for most Dean numbers. By increasing the aspect to d/h = 5, as is shown in Figure 5c-(III), the range of high values of the Dean number becomes smaller and the curve shape changes in comparison to the lower aspect ratios [65]. In Figure 5c, the red dots represent the secondary Dean vortices that appear after the critical Dean number, $De_{c}$. In the cases shown in Figure 5c, the critical Dean number is changing from 30 to 36 and then 40 by increasing the aspect ratio, d/h from 5/3 to 5/2, and 5, respectively. From Figure 5c-(II) to (III) it can also be observed that the projected area of the secondary vortices becomes larger with the increasing aspect ratio [65].

**Figure 6:**
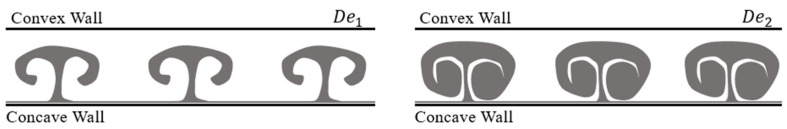
Dean vortices’ structures in a channel with a high aspect ratio, d/h,  of 40, after [74].

Although increasing the aspect ratio leads to an increase in the strength of the vortices, such as what shown in Figure 5a, other numerical and experimental studies show that, for extremely high aspect ratios, 8 < d/h < 40, the primary counter-rotating Dean vortices are converted to multiple pairs of counter-rotating vortices [74,79]. The number of these counter-rotating vortices depends on the Dean number, *De,* and aspect ratio, d/h  [74,79]. For a very high aspect ratio, d/h = 40, in a curved channel, as shown in Figure 6 where $De_{1}<De_{2}$ [74], it can be observed that multiple Dean vortices are formed beside each other. The formation of these vortices starts with the appearance of multiple mushroom shapes [74].

**Figure 7:**
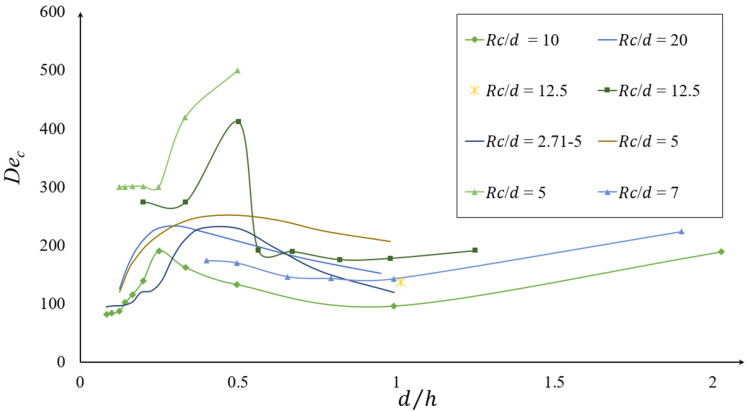
Evaluation of the critical Dean number, $De_{c},$ with respect to the aspect ratio of the channel, *d/h*, for various curvature ratios, $d/R_{c}$ [79].

The aspect ratio of microchannels also influences the formation of secondary Dean vortices and their behavior [14]. Figure 7 shows the evolution of the critical Dean number, $De_{c},$ with the changing of the aspect ratio, d/h. In this figure, the effect of the curvature ratio is obtained from different numerical and experimental works which sit in the range of 2.71 ≤ $R_{c}/d$ ≤ 20 [43,55,79,93,94,95]. From this figure and for $R_{c}/d=$ 10, it can be observed that for the aspect ratios within the range of 1/12 ≤d/h˂ 0.125, the critical Dean number, $De_{c}$, is almost constant. When decreasing the aspect ratio in the range of 0.125 ≤ d/h ˂ 0.25, the $De_{c}$ increases from a local minimum to its maximum, and by aspect ratio 1 it reaches a local minimum. By further increasing the aspect ratio to 1 ≤ d/h ˂ 2, the $De_{c}$ shows a slight increase. The same trend can be seen for $d/R_{c}=$ 12.5 and 7. For $d/R_{c}$ ˂ 7 and $d/R_{c}$ > 12.5, it can be seen that the critical Dean number increases to its maximum and then reduces. This shows the variation in the De with the aspect ratio changes, without a clear correlation between them.

### 3.2. The Effect of the Cross-Section Shape

Several cross-section shapes, such as circular [75,96,97,98,99,100,101,102], triangular [103,104], elliptical [83], and trapezoidal [13,105], have been studied, in addition to rectangular curved channels, to understand the effect of the cross-section shape on the properties of the Dean flow. For these cross-section shapes, the effect of the shape has been investigated regarding their applications in a microchannel, such as particle sorting [76,106,107], micromixing [82,89,108], and heat transfer [93,95]. Table 4 includes a list of the different cross-sectional shapes (of curved microchannels) and the range of De investigated for each case. These cross-sections are used to study the flow behavior and the effect of geometry on the efficiency of the application.

**Figure 8:**
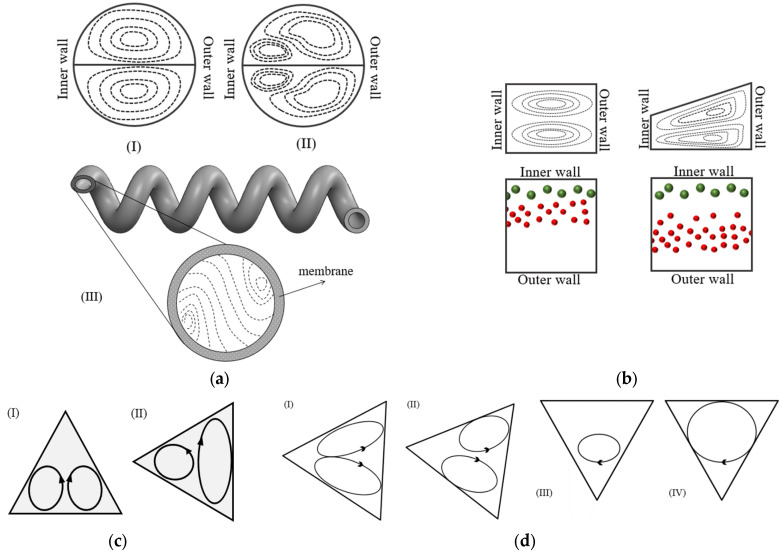
(**a**) Dean vortices in a circular cross-section: (I) for  De < $De_{c}$, (II) for De > $De_{c}$ [64], and (III) in a helical membrane contractor; [109] (**b**) Dean vortices and particle separation equilibrium position in rectangular cross-section in comparison to a trapezoidal cross-section channel with different dimensions [105]; (**c**) differences between the Dean vortices in top-ward and left-ward cross-sections; and (**d**) the Dean vortices in four twisted cross-sections after [103].

The literature review shows that the cross-section can affect the Dean vortices. For a channel with a square cross-section, the general shape of the vortices is similar to the ones in a circular cross-section, as seen in Figure 8(aI,aII). This figure shows the pattern of vortices inside a circular channel for a low and a high De number [64]. In this scenario, the flow topology contains two counter-rotating vortices for De < $De_{c},$ as shown in Figure 8(aI), while a combination of two counter-rotating vortices and two secondary vortices are observed for De > $De_{c},$ as depicted in Figure 8(aII). For a circular cross-section, Siggers and Waters [64] used a numerical approach and showed that the critical Dean number, $De_{c}$, is from 5 to 10 times larger than for a square cross-section channel under similar operating conditions. Chen et al. [109] used the helical membrane contractor channel with circular cross-sections shown in Figure 8(aIII). It can be seen that the Dean vortices are smaller and closer to the walls in comparison to a basic channel without a membrane. In these type of channels, the Dean vortices are continuously renewing the boundary layer, which leads to a consistent mixing of solutes throughout the liquid within the channel. By counteracting concentration polarization, this refreshed boundary layer significantly enhances the overall effectiveness of the liquid–liquid extraction process. 

The ability to manipulate Dean vortices is an important factor that is limited in symmetrical cross-sections [110]. With a trapezoidal cross-section, Dean vortices can be controlled without active intervention [105]. In such cross-sections, either the convex wall or concave wall can serve as the smaller side, and this choice will have distinct effects on the flow behavior. Wu et al. [105] conducted an experiment that showed that, for 2 < De < 22, changing the cross-section from a rectangle to a trapezoid by increasing the size of the concave wall makes the center of the Dean vortices move toward the concave wall, as shown in Figure 8b. For a particle sorting application, this leads to an increase in the space between the settling position of the particles in the channel, making it a better choice for this application [104,105].

The triangular cross-section is another shape that has been investigated in a limited number of studies. Filimonov and Sorvari [103] investigated the Dean vortices inside a serpentine microchannel with a triangular cross-section by performing numerical simulations. For Dean numbers of *De* = 38, 76, and 114, they showed that two symmetrical counter-rotating vortices form, as is shown in Figure 8(cI) [103]. With a 90° rotation of the cross-section, as can be seen in Figure 8(cII), the two symmetric counter-rotating Dean vortices become asymmetric for the same range of Dean numbers [103]. It was shown that the leftward cross-section enhances the heat transfer in comparison to the upward cross-section [103]. This enhancement in the heat transfer can be accounted for by the better mixing due to the more powerful vortices observed in the channel [103]. Twisting the cross-section along the channel also leads to the formation of different patterns of Dean vortices, as can be seen in Figure 8d. Although these different patterns of Dean vortices enhance the mixing, the enhancement is not significant [103].

**Figure 9:**
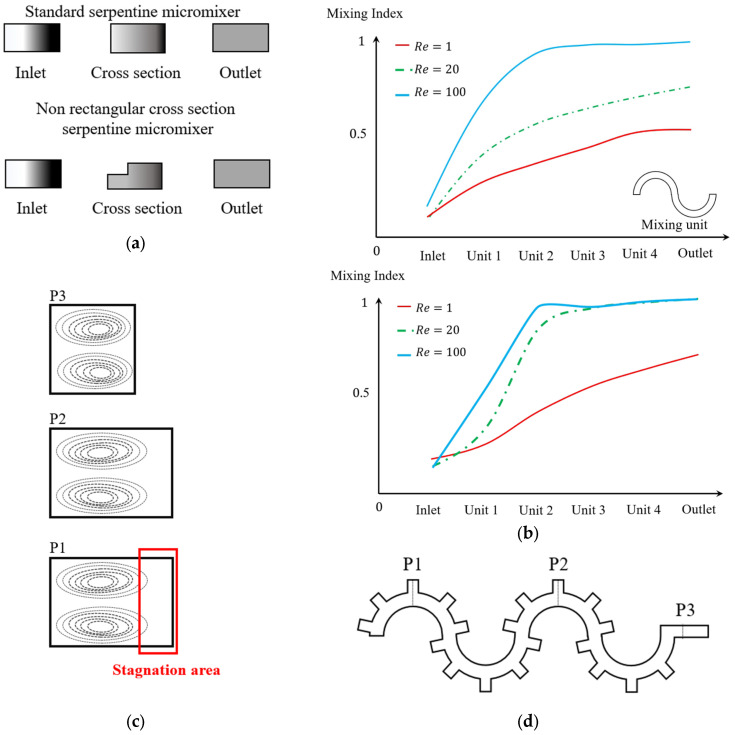

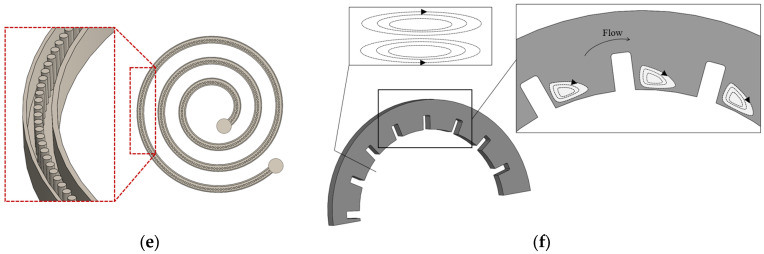
Uncommon cross-sections in curved microchannels. (**a**) Mixing comparison in serpentine channels with a rectangular and multi-rectangular cross-section, with *Re* = 100, after [111]. (**b**) The increase in mixing performance in different sections for *Re* = (1, 20, 100) [111]. (**c**) The effect of changing cross-section on the vortices in a serpentine channel with grooves, after [99]. (**d**) The geometry of a serpentine channel with grooves added to enhance the mixing efficiency, after [99]. (**e**) Curved microchannels with micropillar structures, after [114]. (**f**) Ultra-low-aspect-ratio indented spiral generating multi-vortex, after [116].

In addition to the microchannels with normal cross-sections, microchannels with uncommon cross-section shapes have also been employed and studied [43,111]. The mixing in a microchannel with an incomplete rectangular cross-section has been experimentally investigated for three Reynolds numbers of 1, 20, and 100 [111]. This microchannel was designed to enhance the mixing efficiency in the different units of the serpentine channel and was compared with the standard serpentine microchannels shown in Figure 9a. The comparison of the image intensities for the two geometries under different flow rates is shown in Figure 9b. This indicates that mixing is almost accomplished by the second unit in the notched rectangular cross-section. However, in the conventional rectangular cross-section, this is not expected until the final unit [111,112]. The sudden increase in the mixing index after the first unit is shown in Figure 9b for the notched rectangular cross-section. It indicates that the chaotic behavior of the flow inside this cross-section may contain extra flow structures in addition to the Dean vortices [111,113]. The additional flow structures that are generated in the flow pattern due to the interaction between the flow and geometry are known as secondary vortices, which should not be confused with the secondary Dean vortices [51].

Figure 9c shows the flow visualization in three sections of a serpentine microchannel with a changing rectangular cross-section using grooves, as shown in Figure 9d. The visualized planes on Figure 9c are shown in the microchannel diagram, including the normal section (plane 3), the section with a groove on one side in unit 4 (plane 1), and unit 5 (plane 2). Comparing plane 3 with plane 1 and 2, it can be understood that the Dean vortices become larger, similar to what occurs in a rectangular cross-section with a higher aspect ratio [99]. A noticeable difference is in the stagnation area, which is again not occupied by the Dean vortices [99]. 

A variable unconventional cross-section can be also introduced by adding micropillar arrays in curved microchannels, as shown in Figure 9e. Micropillar arrays, in general, alter the organization of the flow by enhancing its complexity [114]. Particularly, the uneven splitting of laminar streamlines around the micropillar arrays results in size-dependent and predictable trajectories for particles [114]. These structures demonstrate enhanced particle manipulation through the asymmetric bifurcation of laminar streamlines around the micropillar arrays [114]. The advantages include enhanced separation efficiency and a substantial reduction in the required sample volume in the inner channel [115]. Despite these discernible benefits, investigations into their specific interaction with Dean vortices remain limited [115]. Figure 9f shows the schematics of the multi-vortex structures generated in an ultra-low-aspect-ratio spiral with fins. The generated flow structure contains helical vortices at the corner of the fins in addition to the Dean vortices [116]. This multi-vortex flow structure can be adjusted for variety of applications [116].

### 3.3. The Effect of the Curvature Path

Microchannel geometries are made from different combinations of a curved channel, which is the basic structure of a curved microfluidic device [104]. The centerline of the channel generates a path, which is referred to as the curvature path [104]. The curvature path is important since it can increase or decrease the strength of the Dean vortices [13,91,117] and it can also generate more complex flow structures [39]. The radius or direction of the curvature for each of the base segments dictates its flow behavior [104]. The most common path lines have either varying radii of curvature, such as a spiral, or changing curvature directions, such as serpentine [87,118]. The geometries excluded from these categories are considered to have an unconventional path line [97]. 

#### 3.3.1. The Variation of the Radius of Curvature

A microchannel with a variation of the radius of curvature of its channel path, a spiral microchannel, has been used to improve particle sorting and mixing performance [91,119]. In a spiral microchannel, the flow enters from one of the ports at either the center of the spiral or on the outer revolution of it [91,107]. When the flow enters the spiral microchannel from the center port, the flow moves toward the outer revolution, where a general increase in the radius of curvature leads to a decrease in the Dean number [65]. As a result, the strength of the generated Dean vortices decreases as the fluid moves towards the outer revolution [65]. When the flow enters the spiral from the port on the outer revolution, the radius of curvature decreases [91]. In this scenario, the strength of the Dean vortices will increase as they move towards the center of the spiral microchannel [91]. By increasing the Dean number towards the center of the microchannel, there is a possibility of reaching *De_ct_*, indicating the generation of four Dean vortices [91].

**Figure 10:**
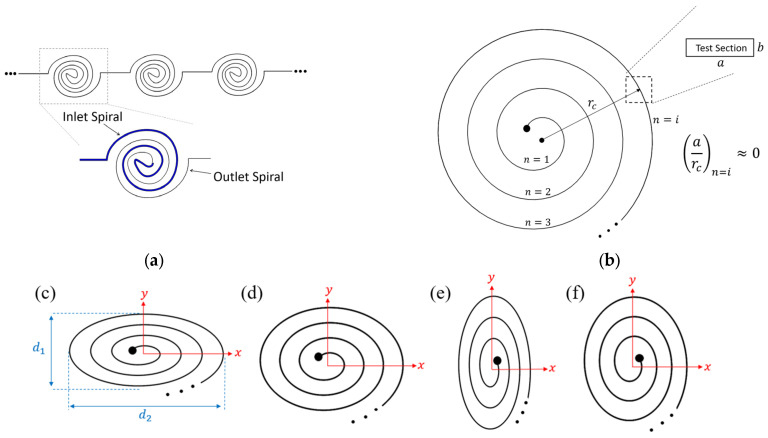
(**a**) Multi-stage spiral microchannel showing three stages of the microchannel and the inlet and outlet spiral part of a single channel [91]. (**b**) The radius of curvature varies in spirals based on the number of the revolutions, from one to infinity. The high radius of the curvature of the larger revolutions decreases the local Dean number [120]. (**c**–**f**) Elliptical spirals with different aspect ratios.

For spiral microchannels, by increasing the number of revolutions, the radius of curvature and the curvature ratio increase. As a result, for a high number of revolutions, the formation of Dean vortices will be suppressed due to the high curvature ratio [120]. In this scenario, to prevent a significant decrease in the Dean vortices’ power, a combination of several successive spiral microchannels with a fewer number of revolutions, Figure 10a, can be used [91]. Considering the same length of the channel, using the multistage spiral microchannel results in more powerful Dean vortices in comparison to a single spiral with a higher number of revolutions [91]. Additionally, in a multiple spiral system, multiple inlets can be added to each stage in order to inject a new phase, e.g., droplets and particles, into the system at each stage [121]. This can be used in many applications, such as generating a linear concentration gradient to enhance drug performance prediction [121].

The aspect ratio of the whole spiral channel is another factor affecting the Dean vortices and flow structures [120]. The aspect ratio of the spiral channel is defined as $d_{2}/d_{1}$, where d1 and d2 are defined based on the spiral shape and orientation in Figure 10c. Four different spirals with different aspect ratios of 3/2, 11/9, 9/11, and 2/3 can be seen in Figure 10c–f, respectively. Despite the aspect ratio of the conventional spirals being equal to 1 (non-elliptical spiral), the radius of curvature in these cases varies frequently in each revolution [120]. This variation leads to frequent changes in the strength of the Dean vortices and the lateral velocity in the microchannel [120]. For cases with a high aspect ratio, the difference between the velocity vectors and Dean vortices of the two sections is significant due to the high change in the radius of curvature [120]. However, in cases with a low aspect ratio, the differences in the velocity vectors in the flow field are not noticeable [120]. These four spiral microchannels were designed for particle separation purposes [120]. The results of their studies show that the purest particle separation was obtained for Figure 10c, where the lateral migration velocity is most significant compared to the other cases [120].

#### 3.3.2. Variation of the Direction of Curvature

**Figure 11:**
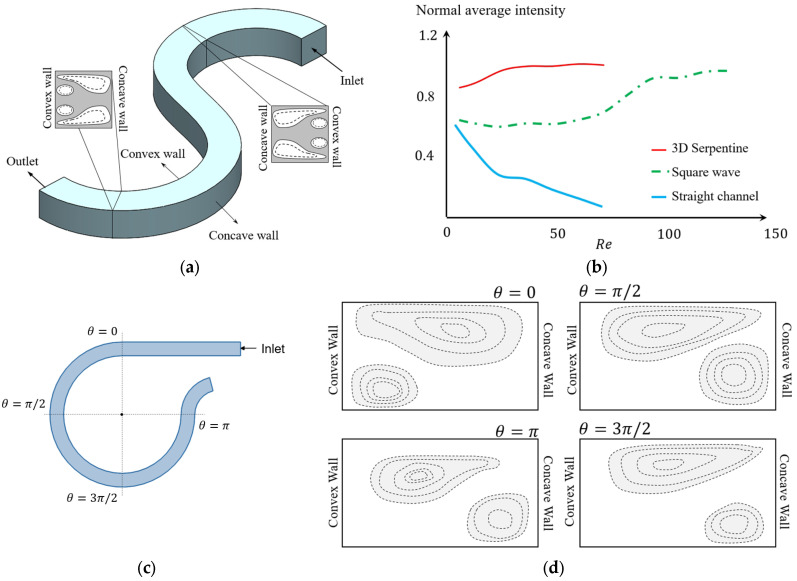
(**a**) Comparing Dean vortices’ topology in two sections from two opposite bends of a serpentine channel, after [89]. (**b**) Comparison between the mixing intensity of three different types of microchannel: serpentine, wavy square, and straight [89]. (**c**,**d**) Dean vortices’ schematics in different sections in a unit of the serpentine channel, after [117].

For serpentine microchannels, unlike the spiral, the radius of curvature is constant. However, the direction of the radius changes for each revolution. This means that the concave wall and convex wall frequently interchange and the pattern of Dean vortices evolve according to the direction of the curvature [89]. Multiple studies have investigated the benefits of flow patterns inside a serpentine channel, as shown in Figure 11a, for applications such as mixing [111,112,117], particle sorting [106,122], and encapsulation [123,124]. Figure 11a shows two sections from two revolutions of a serpentine channel with different curvature directions. The concave wall and convex wall change place in two sections, as can be observed in the zoomed-in views, and the Dean vortices appeared in the same locations with respect to the convex wall [89]. The maximum velocity moves laterally to a point closer to the concave wall. As a result of the changes in direction, the maximum velocity location changes after the transient section to the next curvature, and the core of the Dean vortices relocates [89]. The idea behind using this types of channel is that the transient section, in addition to the Dean vortices, provides a good mixing opportunity [80]. This can be inferred from the results of Figure 11b, which shows the average mixing intensity as an indicator of the mixing efficiency for a straight channel, square wave, and a 3D serpentine. For Reynolds numbers in the range of 0 < *Re* < 80, it can be seen that the average intensity is more than 80%, which is higher than other investigated geometries [112]. 

Four sections of a serpentine segment are shown in Figure 11c with different view angles [117]. On the first velocity field, at θ=0, the pattern of the vortices is completely different from what can be seen at θ=π2. This indicates that a transient section exists in the first quarter of this revolution [117]. From the other sections in Figure 11c, it can be understood that as the flow moves further inside the revolution the Dean vortices form [117]. Figure 11d also shows the configuration of the vortices on each of these sections. Although the mixing performance in geometries with curvature direction variation has been studied in many works, the evolution of the Dean vortices that effect the mixing performance in this geometry has not been investigated, to the knowledge of the authors. 

#### 3.3.3. Unconventional Paths

The channel centerline shape can affect the strength of the Dean vortices or change the pattern of the flow inside the channel to make new phenomena or even generate chaos [111]. Adding a divider or joint to the channel can easily disturb the flow pattern [111]. Therefore, unconventional geometries are sometimes used to enhance the performance of the microchannels through changing the microchannel centerline shape [97].

**Figure 12:**
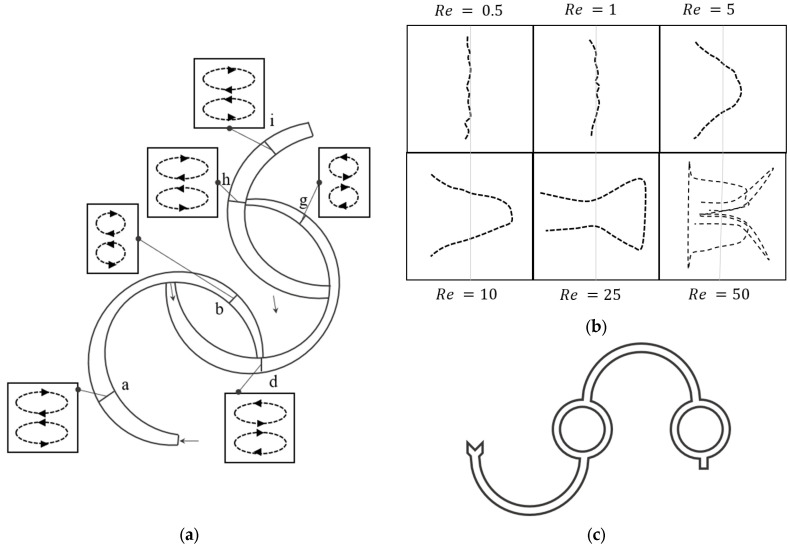
(**a**) An unconventional 3D path designed for micromixers to use the power of Dean vortices [97]. (**b**) The comparison of the interfacial lines for a single curved micromixer and the complex geometry for Re numbers of 1, 5, 10, 25, and 50, after [97]. (**c**) Instances have been reported in which the channel’s design has induced the creation of additional secondary flow patterns alongside the typical Dean vortices. (**c**) shows an unconventional curved channel containing loops between the sections. In this case, when entering the loops, the flow will experience a stagnation point, after [39]. The complexity of the flow structure increases the mixing efficiency. The axis of the vortices generated in this channel is normal to the axis of the longitudinal Dean vortices, after [39].

Figure 12a shows a microchannel geometry along with the Dean vortices and velocity vectors at different sections of the microchannel, obtained through the numerical research undertaken for such an unconventional path with multiple bends [97]. The two flow streams are introduced to the channel, shown by the blue and red colors in the figure. Mixing has been studied for Reynolds numbers in the range of 0.5 <Re< 50. Different directions, sizes, and power of the Dean vortices have been observed at different cross-sections of this channel [97]. The Dean vortices split and their recombination enhanced the performance of the micromixer significantly [97]. It can be seen in Figure 12a that, after joining two different flow streams from different curvatures, the direction of rotation changes to follow the curvature of the channel. For different sections from a to i, with different channel dimensions, the curvature of the channel determines the direction of the rotation, not the maximum velocity or flow rate of the upstream flow. For instance, looking at the vortices before and after section h, shown in Figure 12a, it can be seen that direction of the Dean vortices changes from g to h, but remains constant from f to h. This behavior was considered to be related to the unconventional path of the flow [97]. 

Figure 12b shows the effect of complex geometry on the mixing interfaces for the geometry shown in Figure 12a. By changing the geometry, the shape of the interfacial lines between two fluids in a micromixer changes [97]. The pattern of the interfacial lines is associated with the existence and number of Dean vortices. At lower Reynolds numbers the Dean vortices are not observable. For Re=25 in Figure 12b, the interface line separates near the wall and this can be explained via the existence of Dean vortices. At higher Reynolds numbers, such as 50, the interfacial line deformation indicates the existence of two secondary Dean vortices. Figure 12c also shows another unconventional path that generates additional flow structures in the curved microchannels used for mixing [39].

## 4. The Liquid–Liquid Two Phased Flow in Curved Channels

Droplet-based microfluidics is attracting extensive attention due to its great potential in research and medical applications. A common type of such devices are microfluidic systems that contain two immiscible fluids [125]. This system contains a main fluid flow in a microchannel known as the continuous phase and a second phase with capsules inside it, known as the dispersed phase [126,127,128]. There are many applications in areas such as medicine, biology, and material science that employ the potential of a two-phase flow in microfluidic devices. 

Using such devices, a well-defined environment can be provided for the investigation of an isolated cell. Biochemical reactions can be contained in a droplet to reduce the heat emission or processing time of the reaction and to fabricate materials with special properties. A droplet flowing in a curved channel is also one of the most common passive techniques used to enhance mixing inside a droplet [118]. In applications such as the encapsulation of cells and drug delivery, the shear stresses formed due to the vortices inside the droplets can, in some conditions, damage the living cells [24]. The deformation of the capsule and its internal flow topology affect the performance of microfluidic devices or the procedures they are used in. For this purpose, several studies have investigated the effect of geometry on the topology of droplets and bubbles [129,130,131,132,133,134,135,136] and these are reviewed in this section.

### 4.1. Physics of the Flow Inside a Droplet in Straight Microchannels

**Figure 13:**
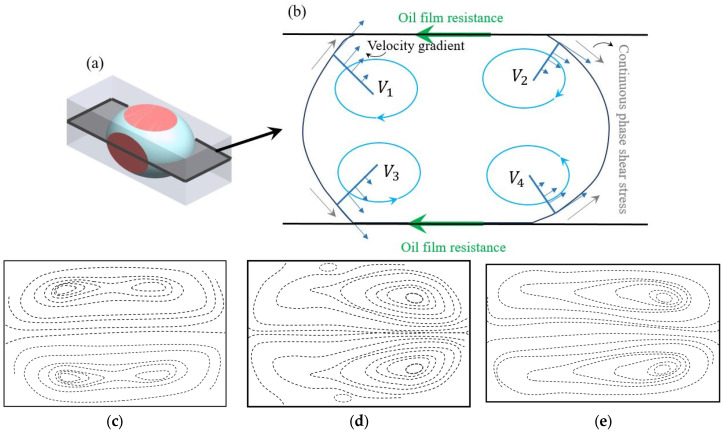
(**a**) Schematic of the forces applying to a droplet flowing in a straight channel. (**b**) Schematic of the flow structure inside the droplet on a cross-section shown from the blue plane, plotted after [137]. Streamlines of the flow inside the droplet at the center plane with the flow rate of (**c**) 0.5 μL/min, (**d**) 5μL/min, and (**e**) 20 μL/min after [137] were obtained experimentally.

To better understand the effect of Dean vortices on a droplet in a curved channel, it is important to briefly review the internal flow topology of a droplet in a straight channel. Figure 13a represents the schematic of a droplet moving in the *x* direction in a microchannel where the droplet size is of the order of the channel width [137]. The purple areas on the 3D schematic of the droplet in Figure 13a indicate the contact surfaces between the channel wall and the droplet. In this figure, it is assumed that the channel is rectangular, as a result, the droplet has a higher contact area at the top and bottom and a lower contact area at the side walls [137]. In the contact area, the droplet is subjected to stress from the walls, which is shown by the yellow arrows of oil films in Figure 13a. This stress is resistance to the droplet movement against the flow direction. In addition to the contact area stress, the continuous phase applies stress to the droplet, which is shown via the dark blue arrows in Figure 13a [137]. 

The flow topology schematic of the cross-section at the middle plane of the droplet is shown in Figure 13b. The superposition of these two stresses forms four different eddies inside the droplet. On each half of the droplet, there are two pairs of counter-rotating vortices, V_1_–V_3_ and V_2_–V_4_. The rotation direction of each vortex is shown with blue arrows in Figure 13b [137]. 

Under the effect of these stresses in lower flow rates (0.5 μL/min), four vortices exist inside the droplet, which is shown in Figure 13c through the contours of the flow streamlines. By increasing the flow rate (5 μL/min) ten times, the vortices on the back side of the droplet start to move forward and toward the edges of the droplet, as shown in Figure 13d [137]. As seen in Figure 13e, the growth of the front side vortices leads to the elimination of the two counter-rotating vortices on the left-hand side [138,139,140] at the higher flow rate (20 μL/min). The change in the velocity distribution is perhaps the reason for the change in the number and shape of the flow vortices [137].

### 4.2. Physics of the Flow Inside a Droplet in Curved Microchannels

**Figure 14:**
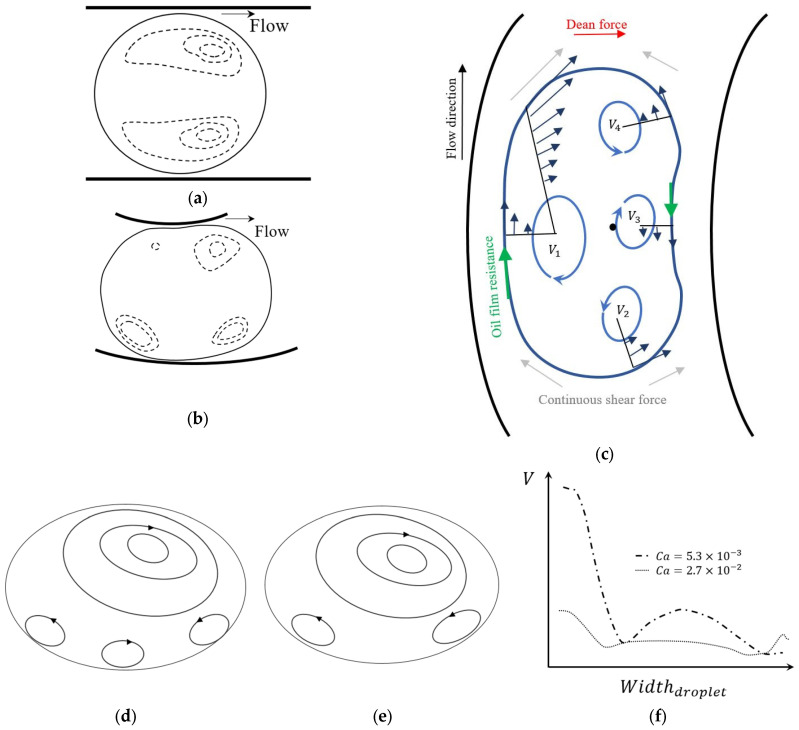
(**a**) Schematics of the flow inside a droplet in a straight microchannel. (**b**) Schematics of the flow inside a droplet entering a curved microchannel, after [141]. (**c**) Schematic diagram of the forces in the middle plane of a droplet in a curved microchannel, after [142]. Vortices inside a droplet in a curved microchannel with (**d**) Ca  = 5.3 × 10^−3^ and (**e**) 2.7 × 10^−2^. (**f**) Variation of the velocity magnitude along the width of the droplet, after [143].

The flow topology of a droplet inside both a straight channel and in a curved channel are compared using the experimental results shown in Figure 14 [141]. The internal flow velocity vectors and magnitude in the straight section of the microchannel are shown in Figure 14a. These are representing the same physics as Figure 13e, with a pair of counter-rotating vortices. The flow structure of a droplet entering the curved channel can be seen in Figure 14b. When the droplet enters a single curved channel attached to the straight channel, the configuration of the vortices changes due to the asymmetric velocity gradient [141]. The higher velocity near the concave wall causes a shift in the vortices’ location toward the sides and the wall [141]. Close to the convex wall, the two vortices proceed to the middle and become larger with a lower velocity [141]. It is worth noting that the number of vortices also increases to four, similar to what is seen with a droplet inside a low continuous phase flow rate. However, with multiple curves or longer curves, the topology of the droplet significantly changes and the vortices are not symmetrical with respect to the centerline of the channel [141]. The maximum velocity position moves toward the concave wall due to the curvature effect. Thus, the relocated maximum velocity affects the symmetry of the flow inside the droplet and changes the configuration of the vortices [141].

Figure 14c shows the schematic of a droplet’s movement inside a curved channel. In this figure, comparable to Figure 13b, the diagram of the forces acting on the droplet is illustrated, as well as the velocity profiles inside the droplet. As is shown in this figure, near the concave wall a clockwise vortex (*V*_1_) which is larger than the others forms. This vortex is shaped in the higher velocity area of the channel. Near the convex wall, three vortices appear in the curve, two counterclockwise (*V*_2_ and *V*_3_) at the sides and a clockwise vortex in the middle (*V*_4_). Similar to the droplet in the straight channel, the topology of the eddies will change with increasing of the continuous phase velocity [142]. The clockwise vortex, *V*_4_, from Figure 14c, disappears at high velocities while the other two vortices on the side that are moving counter-clockwise, i.e., *V*_2_ and *V*_3_, become larger [142].

The interfacial tension between the continuous and dispersed phase is an important factor influencing the physics of the flow in curved microchannels. This factor can be investigated using the capillary number,$\;Ca=\frac{V\mu^{2}}{\sigma \mu ’}$ where V is the continuous phase velocity, μ is the continuous phase dynamic viscosity, μ’ is the dispersed phase dynamic viscosity, and σ is the interfacial tension between the continuous and dispersed phase. Figure 14d–f show the results of an experiment conducted by Liu et al. [143], which demonstrated the effect of the capillary number, Ca, on the internal flow of a droplet in a curved channel, where the capillary number was changed by changing the velocity. Figure 14d shows the formation of four vortices at a low continuous phase velocity, similar to what was illustrated in Figure 14c. The three small vortices at the bottom of the droplet are turned into two larger vortices by increasing the velocity, as is shown in Figure 14e. Though the size of the top vortices is the same for both capillary numbers, the vorticity of the one with a higher capillary number is more powerful than the one with a lower capillary number [143]. This can be further verified through Figure 14f, which shows the magnitude of the velocity on the vertical axis of the droplet. 

### 4.3. Effect of Microchannel Geometry on Droplet Topology 

A circular capillary cross-section will distribute the stresses and forces uniformly on the droplet [137]. This uniformity leads to a simple and symmetrical flow pattern inside the droplet. Adding the effect of curvature geometry to the discussion, such a pattern may no longer remain symmetrical. In rectangular cross-sections, due to the existence of corners and corner flow streams, the topology inside the droplet will change to a more complex shape [137].

**Figure 15:**
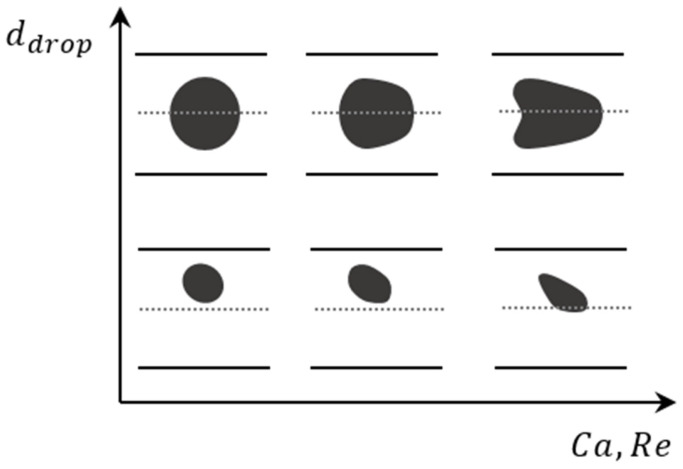
Deformability of droplets in a straight channel with lateral flow inlet, after [147].

The capillary number typically affects the deformability of the droplet [144]. With a small Ca, approximately less than 0.1, the droplet is stiff. The stiffness of the droplet maintains its spherical shape and reduces the effect of the Dean vortices on its internal flow [144,145]. Without changes in the shape of the droplet, the change of the velocity vectors on the boundary is the only parameter that affects its internal flow. Increasing the Ca increases the deformability of the droplet [144,145,146]. A more deformable droplet has a more complex internal flow structure due to the consistent motion of the boundary. Figure 15 shows the effect of the Ca and Re on small and large droplets [143]. It can be seen from the figure that increasing the Ca leads to a completely deformed droplet, where with smaller capillary numbers Ca both small and large droplets remain spherical. This figure shows the deformation of a large droplet and a small droplet [147] at different Reynolds numbers. The schematics of the droplets are shown for their top views in a straight channel, after an experimental study [147].

**Figure 16:**
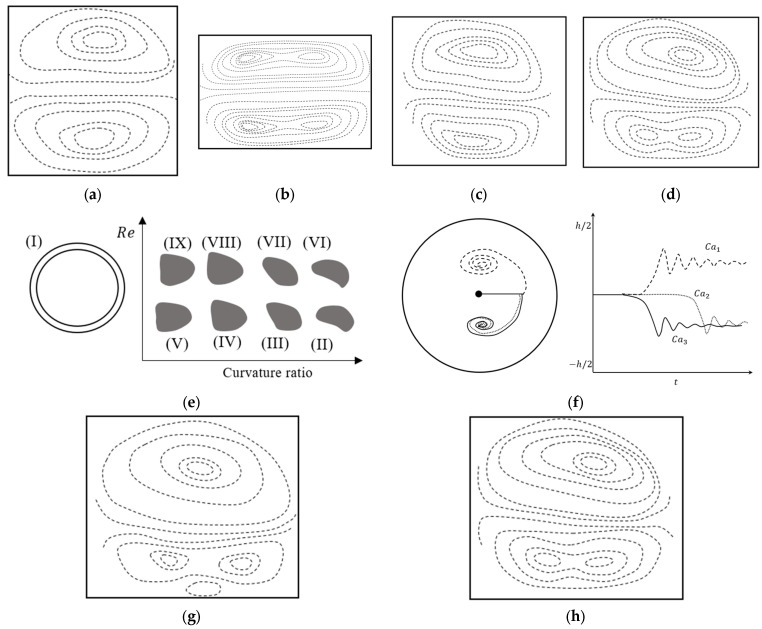
Schematics of the flow inside a droplet moving in a straight channel. (**a**) *β* = 1, after [143], (**b**) *β* = 1.2 [137]. Vortices inside the droplet with (**c**) *β* = 1 and (**d**) β = 1.1 at the same radius of curvature, after [142]. (**e**) Droplet shape with different curvature ratios in a donut-shaped channel and for curvature ratios of (0.05, 0.5, 0.9) and *Re* of 0.04 and 10. (**f**) The trajectory of the droplets with a size ratio less than 0.3 on the cross-section of a curved channel (on the left) and of the channel length (on the right) [145]. Vortices inside the droplet with (**g**) *R* = 2.2 mm and (**h**) *R* = 6.2 mm at the same *β* = 1.1, after [142].

In addition to the channel cross-section shape and capillary number, the droplet size ratio, which can be defined as the ratio of the droplet length to the width of the channel, can significantly affect the topology of the vortices inside the droplet [128,137]. A non-dimensional ratio of the length to width of the droplet, β, which starts from 1 for spherical droplets [137], is used to identify droplet shapes. To study the effect of *β* on the flow topology, an important principle is keeping the capillary number constant [143]. For *β* = 1 in a straight channel, two counter-rotating vortices exist inside the droplet, as is shown in Figure 16a. By increasing *β* to 1.2, the droplet contains four vortices, as is shown in Figure 16b. Two powerful vortices are generated at the front, which is in touch with the continuous phase, and the other two form at the back of the droplet [137,143].

For a curved channel, when β is equal to 1, a similar topology to the straight channel can be observed [137,143]. However, the vortex which is closer to the concave wall occupies more than half of the area [137]. The vortices of the Dean flow are also stronger near the concave wall [137], as shown in Figure 16c. These stronger vortices, which can be seen in both the continuous phase and the droplet, are possibly based on the velocity difference between the concave and convex wall. As is shown in Figure 16d, by increasing the droplet size the vortex closer to the convex wall breaks into two smaller vortices [143]. It is important to note that both droplets shown in Figure 16c,d have the same capillary number. 

Reducing the droplet size to less than 1/2 that of the channel provides us with the ability to study the deformability of the droplet. It can replicate blood cells in a curved vessel [148], or the internal shear rate [149] and flow behavior inside the droplet, which affects the cells in encapsulation [43,150]. The size ratio, ε, can be defined as the ratio of the droplet diameter, a, to the channel diameter, d. For droplets with 0.3<ε<0.6 the droplet starts to form a cone shape, moving forward through the channel as is shown in Figure 16e. Decreasing the curvature ratio to less than 0.3 changes the spherical shape of the droplet into a bean shape [145], as shown in Figure 16e(IV,V,VIII,IX). Increasing the *Re* and decreasing the *Ca* also increases droplet deformability [145].

The small size ratio also affects the trajectory of the droplets in a curved channel [145,146]. Figure 16f shows the trajectory of droplets released on the symmetry plane of the channel where *Re* = 20. The droplets follow a spiral trajectory to its equilibrium position, which is at the center of the Dean vortex. The *Ca* of the droplet determines whether the droplet moves toward the upper or lower vortex. For lower capillary numbers where the droplet is more deformable, it moves through the upper spiral to the center of the upper vortex. However, for a higher Ca where the droplet is stiffer, the motion is toward the lower vortex with the lower spiral. With increasing the capillary number to higher values the droplet remains on the center axis for longer and starts to follow the spiral trajectory further into the channel [145].

The radius of curvature is another important parameter affecting the flow pattern in the curved channel and strength of the generated Dean vortices [142]. This parameter also affects the flow topology inside the droplet [142]. Figure 16g,h show the flow pattern inside a droplet in two different radii of curvature. In both cases *β* is equal to 1.1. As it can be seen from the figure, the number of vortices and their location are the same as the droplet passing through the curved channel with a lower radius. However, the single vortex near the concave wall vanishes in Figure 16h. By decreasing the radius of curvature, the vortex near the concave wall becomes stronger and its area increases, which is similar to increasing the vortices’ power in the continuous phase. Stronger vortices create a stronger shear rate and increase the rotating velocity inside the droplet. Figure 16g shows a droplet with *β* = 1.1 in a channel with a radius of curvature equal to 2.2 mm. Inside the droplet, four vortices are formed on a strong large vortex near the concave wall: two vortices near the concave wall close to the trailing edge and leading edge of the droplet and one vortex close to the convex wall [142]. 

## 5. Conclusions

This review opens doors for those who research the fundamental physics of the flow in curved microchannels and use that physics to control the performance of microdevices. In this review, the physics of the Dean flow and the formation of Dean vortices in curved channels is reviewed. Following the physics, the relationship between the Dean number and the vortices’ configuration is investigated. Several equations have been introduced and used for Dean number calculations by different researchers. The variation in the calculation of this dimensionless number, as a common reporting parameter, can lead to confusion in attempts at its interpretation and, following that, in employing the reported information in other studies. Therefore, the most common versions of this equation are presented in this work. The equations are categorized into two groups, numerical and experimental equations, depending on the type of research each equation is dominantly used in. 

The effect of different geometrical properties on the Dean vortices and their applications is also reviewed. Various cross-sections, including circular, triangle, square, rectangle, and trapezoid, are employed in microchip designs to manipulate flow structures. In rectangular channels, a wide range of channel aspect ratios, and their effect on generation of different configurations of Dean vortices is investigated. In high aspect ratios, multiple pairs of Dean vortices can be observed in the channel cross-section. The variations in the direction or radius of curvature is also an affecting parameter that has been reviewed in addition to the unconventional three-dimensional channel paths containing multiple curved sections. Each of these properties has been shown to be used as passive manipulation options to change the configuration of Dean vortices. Serpentine channels are used to improve the performance of micromixers. Spiral channels are employed in applications with sorting/separating purposes. 

A review of droplet dynamics under the effect of flow through a curved geometry is reported. A substantial discussion on the internal flow structure of a droplet and changes in the vortices under the effect of a curved geometry is presented. It has been reported that a droplet in a curved channel contains four vortices instead of two. The effect of droplet stiffness, Ca, droplet size, and the radius of curvature, is shown on the configuration of the vortices inside the droplet. Stiffer droplets are less sensitive to their surrounding flow structure. Also, reduced deformability has been reported for droplets with lower Cas. These parameters affect the trajectory in addition to the deformation of a droplet passing through the channel. The direction of the spiral path the droplet takes to its settling point shifts from the upper half to the lower half of the channel with an increasing Ca.

Although there have been extensive investigations on the applications of Dean vortices, the physics of this phenomena is yet to be fully understood. In spite of significant progress, not being able to predict the behavior of the vortices, and their interactions with particles and droplets, can be a major drawback in the design of microfluidic devices employing a curved geometry. As new effective parameters are introduced in this review, based on existing publications, it is expected that, in the future, we shall see more studies analyzing the effect of affecting parameters on Dean vortices and investigating their interactions with particles and droplets. 

## Figures and Tables

**Table 1:** Equations based on mean axial velocity used for Dean number calculations in experimental investigations. In these equations, d represents the hydraulic diameter of the channel, $R_{c}$ is the radius of curvature of the channel, ν is the kinematic viscosity of the fluid,$\;W_{0}$ is the mean axial velocity, δ is the curvature ratio which is defined as the ratio of hydraulic diameter, d, to the radius of curvature, $R_{c}$, and $Q_{c}$ and $Q_{s}$ are also the fluxes for curved and straight pipe, respectively.

	Equations		Author(s)	Description
Velocity-based	$K=2\left(\frac{d}{R_{c}}\right){\left(\frac{dW_{0}}{\nu }\right)}^{2}=\left(\frac{2W_{0}{}^{2}d^{3}}{\nu^{2}R_{c}}\right)$	(2)	Dean (1928)	Study the relationship between mass flux and geometry [47].
	$De_{1}={\left(\frac{1}{2}K\right)}^{1/2}\times \frac{Q_{c}}{Q_{s}}=2^{5/2}\frac{Re}{\sqrt{dR_{c}}}$	(3)	Dyke (1978)	Dean’s series for steady fully developed laminar flow through a toroidal pipe with a small curvature ratio [73].
	$De_{2}=\frac{Re}{\sqrt{d/R_{c}}}$	(4)	Bara et al. (1992)	Laminar Newtonian flow inside a square duct with non-symmetrical geometries [66].
	$De_{3}=2\delta^{1/2}Re={\left(\frac{d}{R_{c}}\right)}^{1/2}\left(\frac{2d\bar{W_{0}}}{\nu }\right)$	(5)	Berger and Talbot (1988)	Study the characteristics of the flow in the curved channel [59].
	$De_{4}=\frac{W_{0}d}{\nu \sqrt{d/r_{i}}}$	(6)	Ligrani and Niver (1988)	Curved channels with a high radius of curvature, riro≅1 [74].
	$De_{5}=Re\sqrt{\frac{d}{R_{c}}}\;$	(7)	Kim and Lee (2009)	3D velocity field inside a circular microtube; d is the inner diameter of the microtube [75].
	$De_{6}=Re\sqrt{\frac{d}{2R_{c}}}$	(8)	Berger et al. (1983) Nivedita et al. (2017), Seo et al. (2012)	Investigate Dean vortices inside a low-aspect-ratio spiral [59,65,76].

**Table 2:** Some of the proposed Dean number definitions based on the pressure gradient, mainly used in numerical investigations. In these equations, d represents the hydraulic diameter of the channel, $R_{c}$ is the radius of curvature of the channel, ν is the kinematic viscosity of the fluid, μ is the dynamic viscosity of the fluid,$\;W_{0}$ is the mean axial velocity, G is the pressure gradient defined as $G=\partial P/\left(R\partial \theta \right)$, and C is the dimensionless constant defined as $Gd^{2}/\left(\mu W_{0}\right)$.

	Equation		Author(s)	Description
Pressure-based	$De_{7}={\left(\frac{2d^{3}}{\nu^{2}R_{c}}\right)}^{1/2}\frac{Gd^{2}}{\mu }=4{\left(\frac{2d}{R_{c}}\right)}^{1/2}\frac{Gd^{3}}{4\mu \nu }$	(9)	McConalogue and Srivastava (1968)	Study the motion of the flow in curved tubes assuming that $\frac{Gd^{2}}{\mu W_{0}}=const.$ [69].
	$De_{8}=\frac{2d^{3}}{\nu^{2}R_{c}}{\left(\frac{Gd^{2}}{4\mu }\right)}^{2}=2\left(\frac{d}{R_{c}}\right){\left(\frac{Gd^{3}}{4\mu \nu }\right)}^{2}=\frac{G^{2}d^{7}}{8\mu^{2}\nu^{2}R_{c}}$	(10)	Burger and Talbot (1988)	Study the flow characteristics in a curved channel with a constant fully developed flow [59].
	$De_{9}=\frac{2d^{3}}{\nu^{2}R_{c}}{\left(\frac{Gd^{2}}{\mu C}\right)}^{2}$	(11)	Burger and Talbot (1988)	Study characteristics of the flow in a curved channel [59].
	$De_{10}=\frac{Gd^{3}}{16\mu \nu }{\left(\frac{d}{R_{c}}\right)}^{1/2}$	(12)	Norouzi and Biglari (2003)	Analytical solution for the flow in a curved channel for low Dean numbers [77].
	$De_{11}=\frac{Gd^{3}}{\mu \nu }{\left[\frac{2d}{R_{c}}\right]}^{\frac{1}{2}}$	(13)	Howell et al. (2004)	Experimental study of the Dean vortices in a micromixer. G is the pressure gradient on the channel centerline [78].

**Table 3:** Single curve and spiral microchannels’ information used to study the initial and critical Dean numbers’ relation to the number of revolutions [55,65,77,80,82,83,84,85,86,87,88].

Case	*AR*	*d*	*h*	*R_c_*	*De_i_*	*De_c_*	Cross-Section	Channel	Ref.
1	1.60	250	150	various	28.5	30.5	rectangle	spiral	[52]
2	2.5	250	100	various	34.5	37	rectangle	spiral	[52]
3	5	500	100	various	31.5	40	rectangle	spiral	[63]
4	2.5	250	100	various	NA	45	rectangle	spiral	[59]
5	3.3	200	60	2000		23	rectangle	spiral	[75]
6	0.25	NA	NA	NA	NA	72.5	rectangle	curved	[77]
7	0.5	NA	NA	NA	NA	70	rectangle	curved	[78]
8	1	NA	NA	NA	NA	67.5	rectangle	curved	[79]
9	2	NA	NA	NA	NA	60	rectangle	curved	[80]
10	4	NA	NA	NA	NA	52.5	rectangle	curved	[81]
11	2	NA	NA	NA	NA	100	rectangle	curved	[82]
12	1	NA	NA	NA	NA	45	rectangle	curved	[52]
13	0.5	NA	NA	NA	NA	65	rectangle	curved	[52]
14	0.3	NA	NA	NA	NA	80	rectangle	curved	[63]
15	0.25	NA	NA	NA	NA	95	rectangle	curved	[59]
16	0.2	NA	NA	NA	NA	70	rectangle	curved	[75]
17	0.17	NA	NA	NA	NA	57.5	rectangle	curved	[77]
18	0.14	NA	NA	NA	NA	50	rectangle	curved	[78]
19	0.125	NA	NA	NA	NA	42.5	rectangle	curved	[79]
20	0.1	NA	NA	NA	NA	40	rectangle	curved	[80]
21	0.08	NA	NA	NA	NA	40	rectangle	curved	[81]
22	1	NA	NA	NA	NA	72.5	rectangle	curved	[82]
23	1.25	NA	NA	NA	NA	95	rectangle	curved	[52]
24	1	NA	NA	NA	NA	87.5	rectangle	curved	[52]
25	0.8	NA	NA	NA	NA	87.5	rectangle	curved	[63]
26	0.7	NA	NA	NA	NA	92.5	rectangle	curved	[59]
27	0.55	NA	NA	NA	NA	95	rectangle	curved	[75]
28	0.5	NA	NA	NA	NA	204	rectangle	curved	[77]
29	0.3	NA	NA	NA	NA	135	rectangle	curved	[78]
30	0.2	NA	NA	NA	NA	137.5	rectangle	curved	[79]
31	1.7	250	150	2000	17	206	rectangle	curved	[80]
32	2	30	15	400	20	NA	rectangle	curved	[81]
33	1	150	150	4000	6.8	100	rectangle	curved	[82]
34	1	200	200	5000	10	NA	rectangle	curved	[82]
35	1	200	200	2500	0.316	NA	rectangle	curved	[82]

**Table 4:** Different cross-section shapes studied in curved microchannel investigations.

Shape		Investigator(s)	Operational De
Square	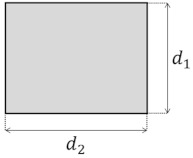	H. Fellouah et al. (2006) [79]	10<De<400
Circle	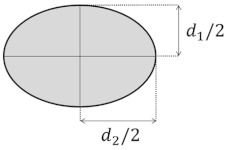	J. Siggers and S. Waters (2005) [64]	10<De<15,000
Triangle	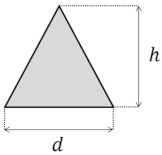	R. Filimonov and J. Sorvari (2017) [103]	17<De<142
Trapezoid	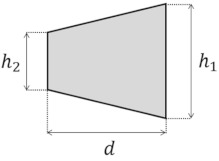	L. Wu et al. (2012) [105]	2.64<De<21.12

## Data Availability

Not applicable.
